# Editorial: New endoscopic techniques for ventral hernia repair

**DOI:** 10.3389/fsurg.2023.1245620

**Published:** 2023-07-26

**Authors:** Gabriel Sandblom

**Affiliations:** ^1^ Department of Clinical Science and Education Södersjukhuset, Karolinska Institutet, Stockholm, Sweden; ^2^ Department of Surgery, Södersjukhuset, Stockholm, Sweden

**Keywords:** ventral hernia, robot-assisted, chemical component separation, transabdominal retromuscular repair, transabdominal preperitoneal repair

**Editorial on the Research Topic** New endoscopic techniques for ventral hernia repair

The rapid development of endoscopic techniques over the last three decades has enabled the safe repair of complex ventral hernias with a smoother recovery then after open ventral hernia repair and with less persisting postoperative pain. Endoscopic ventral hernia repair followed the introduction of the laparoscopic technique in the early 1990's, as with most other laparoscopic procedures. The procedure involved covering the hernia defect intraperitoneally with a mesh i.e., intra-abdominal Onlay Mesh (IPOM) repair, and was shown to have several advantages compared to established open techniques at that time. Early follow-up studies on IPOM repair showed low risk for postoperative complication, low recurrence rates, and high patient satisfaction (1). However, in contrast to open repairs that were mainly based on the onlay or sublay approach, IPOM repair involved placing the mesh within the abdominal cavity. Direct contact with the intestines, however, introduced the risks of adhesions, infection, or fistulae involving the mesh, and new types of mesh had to be developed to reduce those risks.

After two decades’ development, refinement, and numerous attempts to improve the technique, the unavoidable disadvantages of intra-abdominal mesh placement have become apparent (2). The tension caused by the mesh may lead to long-term pain, and even if new compound meshes have improved the situation, intraperitoneal mesh-related complications cannot be entirely avoided. Furthermore, compound meshes required for IPOM repair are more expensive.

In recent years, our understanding of anatomy derived from open hernia surgery has come to use when carrying out endoscopic hernia repair. Technological advances and increased experience have made other approaches to ventral hernia repair possible, entering anatomical areas that were previously only considered accessible with an open technique. The new endoscopic techniques for placing the mesh outside the abdominal cavity include endoscopic Mini- or Less-Open Sublay repair (eMILOS), endoscopic Totally Extraperitoneal Approach (TEA), TransAbdominal PrePeritoneal repair (TAPP), and enhanced-view totally extraperitoneal repair (eTEP). In comparison with onlay repairs, these techniques lead to greater retention strengths (3).

With the TAPP technique, the preperitoneal space is entered through the abdominal cavity, after which the peritoneum is closed over the mesh. Laparoscopic TransAbdominal Retromuscular (TARM) repair is performed through a longitudinal incision in the peritoneum and posterior rectus sheath, providing access to the retromuscular space which enables placement of the mesh in a sublay position.

The development of the new techniques has to a great extent been fueled by the possibilities provided by the new technologies. Nevertheless, the purpose of these innovations should be on an improvement of the long-term outcome and biomechanical properties of the repairs and not on the benefits perceived intraoperatively (4). As for many other cases when a progress in medicine is seen, the goals tend to be defined by the healthcare provider handling the new tools rather than quality measures defined by patient perspectives.

The new approaches are technically demanding, but the development of robot-assisted laparoscopy has overcome many of obstacles that could not be managed with conventional laparoscopy. Robotic assistance facilitates dissection and provides access to all corners of the surgical field. In a study from Germany, eTEP, eMILOS and TAPP were introduced as routine procedures for ventral hernia repair parallel to training in robot surgery at the clinic Tang et al. That study suggested that it is possible to go from open preperitoneal and retromuscular techniques to robot-assisted transabdominal ventral hernia repair placing the mesh in the same anatomical positions. In this context, IPOM repair represents only a transitory era in the development of ventral hernia surgery.

In a study from Sweden, a pragmatic approach was applied to robot-assisted ventral hernia repair where the primary aim was to carry out TAPP or TARM repair while being prepared to convert to IPOM if this was found to be too difficult. The study showed that this strategy was safe and resulted in rapid recovery and a low rate of long-term postoperative pain Lindström et al.

Repair of a parastomal hernia is widely considered a challenging procedure as it requires restoration of fascial strength while not interrupting continuity between the intra-abdominal intestine and the external stoma. However, a case report showed that totally extraperitoneal placement of a polypropylene mesh around the stoma using conventional laparoscopy was possible Jiang et al. This approach avoids the risks associated with intra-abdominal mesh such as with the Sugerbaker technique.

Concurrent with the development of endoscopic techniques, surgical approaches were developed, such as transverse abdominal release, to reduce the tension caused when closing the abdominal wall in very large incisional hernia repair. Recent studies have shown, however, that botulinum toxin injections in the abdominal muscle prior to repair may lead to relaxation of the abdominal wall and reduce the need for surgical component separation. In a case series of patients undergoing IPOM repair or laparoscopic-open-laparoscopic ventral hernia repair, preoperative administration of botulinum toxin was found to be safe, and that it increased abdominal volume and lengthened laterally retracted abdominal muscles Bauer et al.

In many respects, the development of these new endoscopic repair techniques for ventral hernia repair has led to the rediscovery of the same anatomical spaces that were used for open ventral repair prior to the introduction of the IPOM technique. Since these procedures retain functional anatomy using mesh to reinforce the abdominal wall rather than to overbridge the defect, they have more favorable outcomes than laparoscopic IPOM repair, with smooth and rapid postoperative recovery as well as reduced risk for long-term postoperative pain.

The greatest potential benefit from the transfer from IPOM and onlay repairs is probably the improved biomechanical properties from using mesh with sufficient size and placing them in an appropriate position. By providing access to anatomical spaces other than the abdominal cavity or the prefascial space they enable placing the mesh with sizes adapted to the defect size and fixing them in a way that the tissue tension is adjusted to the functional anatomy of the abdominal wall, thereby reducing the risk of persisting pain and recurrences Bauer et al. Future studies should focus on evaluating ventral hernia surgery in terms of biomechanical strength and resistance to the forces caused by cyclic loading of the abdominal wall.
